# Biomarkers of Oxidative Stress for Neonatal Lung Disease

**DOI:** 10.3389/fped.2021.618867

**Published:** 2021-02-18

**Authors:** Giuliana Ferrante, Giuseppe Carota, Giovanni Li Volti, Mario Giuffrè

**Affiliations:** ^1^Dipartimento di Promozione della Salute, Materno-Infantile, Medicina Interna e Specialistica d'Eccellenza “G. D'Alessandro”, Università degli Studi di Palermo, Palermo, Italy; ^2^Dipartimento di Scienze Biomediche e Biotecnologiche, Università degli Studi di Catania, Catania, Italy

**Keywords:** oxidative stress, biomarker, lung disease, newborn, prematurity

## Abstract

The transition from prenatal to postnatal life causes a significant increase in arterial oxygen tension and the activation of metabolic pathways enabling the newborn's adaptation to the extra-uterine environment. The balance between pro-oxidant and anti-oxidant systems is critical to preserve cellular functions. Indeed, oxidative stress (OS) occurs when the production of free radicals is not balanced by the activity of intracellular antioxidant systems, contributing to cellular and tissue damage. Perinatal OS may have serious health consequences during the postnatal period and later in life. Namely, OS has been recognized as the major cause of lung injury in newborns, especially those preterm born, due to their immature lung and antioxidant systems. The development of OS biomarkers has gained increasing research interest since they may provide useful insights about pathophysiological pathways underlying OS-mediated pulmonary diseases in newborns. Moreover, their implementation in clinical settings may help to early identify high risk-newborns and to provide targeted treatment. Ideally, a biomarker should demonstrate ease of use, biological validity and reproducibility, high sensitivity and specificity. However, none of the clinically validated biomarkers so far have been qualified for neonatal lung disease. Additionally, the complex technical procedures and the high cost of such determinations have hampered the use of OS biomarkers in clinical practice. This review aims to evaluate the current evidence on the application of biomarkers of oxidative stress for neonatal lung disease and exploring the most relevant issues affecting their implementation in practice, as well as the associated evidence gaps and research limitations.

## Introduction

The transition from prenatal to postnatal life causes a significant increase in arterial oxygen tension and the activation of metabolic pathways enabling newborn's adaptation to the extra-uterine environment (1). The balance between pro-oxidant and anti-oxidant systems is therefore required to preserve cellular functions.

**Figure 1:**
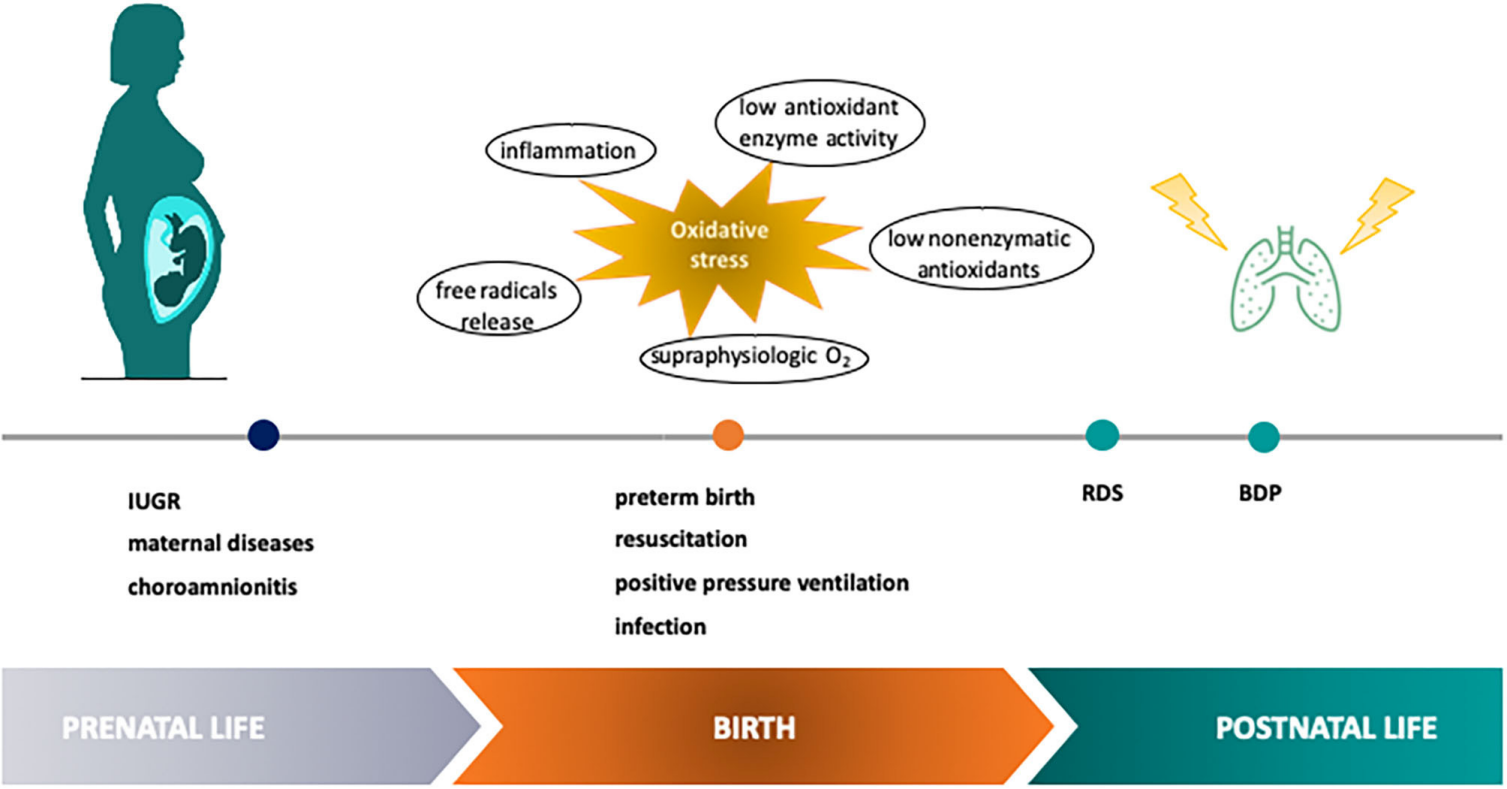
Etiologic factors of OS-mediated neonatal lung injury in the perinatal period. Perinatal OS has been recognized as a point for the convergence of endogenous and exogenous events contributing to lung damage through free radical generation. IUGR, intra uterine growth restriction; RDS, respiratory distress syndrome; BPD, bronchopulmonary dysplasia.

Neonatal lung injury, intended as the acute and/or chronic inflammatory-mediated cellular dysfunction and damage occurring in the lung during the perinatal period, acknowledges a variety of etiologic factors such as genetic, hemodynamic, metabolic, mechanical, and infectious mechanisms, acting in a synergistic fashion. Also maternal conditions and inflammatory placental disorders may play a significant role in the development of lung damage in the perinatal period (2) (Figure 1). Oxidative stress (OS) has been recognized as the final endpoint for the convergence of endogenous and exogenous events, and it is thought to be responsible for cellular, tissue and organ damage through free radicals (FRs) generation (3). FRs are highly reactive compounds able to interact with cellular biomolecules (e.g., proteins, lipids, DNA) producing oxidized derivatives (4). Indeed, OS occurs when the production of FRs is not balanced by the intracellular antioxidant systems, contributing to cellular and tissue damage.

Perinatal OS may have serious health consequences during the postnatal period and later in life, via the modulation of gene expression and cell growth (5). Preterm newborns are particularly susceptible to OS-related conditions with short-and-long-term consequences. In particular, the role of OS has been demonstrated in the most frequent prematurity-related disease, the respiratory distress syndrome (RDS), as well as in the development of bronchopulmonary dysplasia (BPD), which is one of the major causes of chronic respiratory diseases among infants (6). Therefore, the availability of reliable tools to monitor and control OS in the neonatal period would be helpful to avoid its detrimental effects and to improve respiratory health outcomes, especially in preterms (7). In this context, it appears attractive to evaluate OS at a molecular level by means of biomarkers obtained from biomolecules oxidation (8). Indeed, the implementation in clinical settings of metabolite detection in biosamples able to evaluate host susceptibility to OS by measuring lipid, protein, and DNA damage, may help to early identify high risk-newborns, to early diagnose OS-associated disease and to provide targeted treatment.

This narrative review aims to evaluate the current evidence on the application of biomarkers of OS for neonatal lung disease obtained from biomolecules oxidation and explore the most relevant issues affecting their implementation in clinical practice, as well as the associated evidence gaps and research limitation.

We searched original papers in English in the PubMed database using the following keywords, used separately and in combination: biomarker, oxidative stress, newborn, prematurity, lung disease, respiratory distress syndrome, bronchopulmonary dysplasia, pneumonia. Age restrictions were set to newborn (birth −1 month). No limitations were set for the date and study country. We also consulted the reference lists of the retrieved articles. Exclusion criteria included: commentaries, letters, and case reports.

## Inflammation and Oxidative Stress in the Newborn Lung

The imbalance between reducing agents and systems involved in the disposal of FRs or reactive oxygen species (ROS) leads to OS, which has been recognized as the major cause of lung injury in newborns, especially in preterms, due to the immaturity of their lungs and antioxidant systems. The expression of antioxidant enzymes, as well as the availability of the most relevant non-enzymatic antioxidants, is not fully achieved until the end of gestation. In particular, maturation of the antioxidant system generally occurs during the last weeks of gestation enabling the newborn to face postnatal afflux of oxygen to tissues (9). Moreover, preterm newborns frequently require positive pressure ventilation using a gas admixture enriched with oxygen to be successfully stabilized. Such a postnatal increase in oxygen availability contributes to enhance OS (10).

FRs are very high reactive agents that are capable to induce chain reaction, causing cellular dysfunction and damage in cellular components such as lipids, proteins, and DNA at a critical developmental point, leading to high vulnerability to many disorders (11). Hence, to identify biomarkers of FR-mediated diseases may be crucial for their management.

### Biomarkers of Lipid Peroxidation

OS occurs even in the arachidonic acid metabolism, playing a pathogenic role in inflammatory disease (12). Cellular membranes contain poly-unsaturated lipids which are susceptible to oxidation. FRs-induced peroxidative damage to membrane lipids is an event potentially leading to cell injury that, in certain conditions, may result in irreversible damage to the cells. Indeed, lipid peroxidation, as an uncontrolled self-enhancing mechanism, causes disruption of membrane lipids and other cell components. High levels of lipid hydroperoxides (LOOH), malondialdehyde (MDA), and isoprostanes (IsoPs) and isofurans (IsoFs) have been detected in various biological fluids during pregnancy (13). IsoPs and IsoFs are prostaglandin-like compounds produced, respectively from the oxidation of arachidonic acid and docosahexaenoic acid. F2-isoprostanes, an established biomarker for oxidative damage, are a series of prostaglandin F2–like compounds produced independently of cyclooxygenase, as products of the radical-catalyzed lipid peroxidation of arachidonic acid (14). There are many F2-isoprostanes, but the most often measured is 8-iso-prostaglandin F2α (8-iso-PGF2α). The excessive generation of 8-iso-PGF2α has been attributed not only to non-enzymatic or chemical lipid peroxidation but also simultaneously to the activity of prostaglandin-endoperoxide synthase enzymes which are induced during inflammation (15, 16). Increased levels of plasma 8-iso-PGF2α have been demonstrated in newborns during the first week, compared with healthy adults, and have been associated with a greater risk of lung disease (17, 18). An oxygen insertion step diverts intermediates from the IsoPs pathway to IsoFs that contain a substituted tetrahydrofuran ring. Like the IsoPs, they are chemically stable so can act as biomarkers of OS damage (5).

MDA is another biomarker originated by polynsatured fatty acid peroxidation and able to bind proteins or nucleic acids very quickly with a high cytotoxic effect (5).

### Biomarkers of Protein Oxidation

A number of proteins and amino-acidic residues may be prone to oxidative damage by FRs reactions. Given that during the oxidation of proteins, carbonyl groups (-CO=O) are introduced into the side-chains of the proteins, tissues injured by OS generally contain high levels of carbonylated proteins. Therefore, the measure of carbonyl levels is the most commonly used biomarker of OS-related protein damage. The detection of advanced oxidation protein products (AOPPs) in biologic fluids can be another strategy to detect and to estimate the degree of OS-mediated protein damage. AOPPs derive from protein exposure to FRs without oxidant properties and are elevated in hypoxic newborns, especially preterm (19).

### Biomarkers of DNA Oxidation

7,8-hydroxy-2′-deoxyguanosine (7,8-OHdG) is a guanosine base oxidation product, frequently used as a biomarker of OS-related DNA damage. Since oxidative DNA lesions like oxidized nucleosides and bases are excreted into the urine without undergoing further metabolization processes, urinary 7,8-OHdG is considered a reliable biomarker of cellular OS (5).

### Antioxidant Systems

Many well-integrated antioxidant systems exist to counteract and reduce the propagation of FRs, through their scavenging capacity (20). These defenses are divided into low molecular weight antioxidant compounds like lipoic acid, Vitamins A, E and C, and glutathione (GSH) and antioxidant enzymes, like glutathione peroxidase (GPX), superoxide dismutase (SOD) and catalase (CAT) (2, 21). GSH is a thiol and tripeptide, synthesized by glycine, cysteine, and glutamate in the liver, which plays a key role as a vital factor in metabolic protective function related to the reduction of hydroperoxides and the quenching of FRs (22, 23). GSH functions by maintaining a reduced state of the cellular environment, and by removing potentially toxic electrophile molecules, thereby protecting cells from damaging oxygen products (9, 24). The GSH antioxidant system includes a group of functionally related enzymes: GPX, which can reduce hydroperoxides (H_2_O_2_) using GSH as substrate; glutathione reductase, which reduces oxidized glutathione restoring the GSH; and glutathione S-transferase, capable to reduce peroxides and conjugate several toxic electrophiles compounds to nucleophilic GSH (25, 26). SOD is considered the most relevant antioxidant in the cell. It catalyzes the dismutation of O–superoxide anion to H_2_O_2_ and molecular oxygen (O_2_). However, SOD acts also as a pro-oxidant, producing H_2_O_2_; therefore, an imbalance between SOD and antioxidant enzymes such as GPX and CAT could be dangerous (27). CAT is a common antioxidant enzyme, located primarily in the peroxisomes, that catalyzes the conversion of H_2_O_2_ into H_2_O and O_2_ (27).

## Oxidative Stress and Neonatal Lung Disease

### RDS

RDS is the commonest respiratory disorder and a leading cause of mortality and morbidity in preterm newborns. RDS is characterized by reduced alveolar volume, immature extracellular matrix, decreased compliance of the lungs and pulmonary edema due to loss of the integrity of the alveolocapillary barrier, associated with impaired surfactant production and function. Overall, these changes end into impaired gas exchange and increased tendency of the alveoli to collapse. Surfactant deficiency has been recognized as the most relevant factor in the pathogenesis of RDS in very preterm and moderately preterm newborns, while cesarean section and lung infection seem to play a major role in late preterms with RDS (28). Although the mechanisms of lung injury in RDS still need to be fully elucidated, it has been suggested a role of OS in the physiopathology of this lung disease. Indeed, OS appears to be related both to conditions predisposing to RDS (e.g., gestational diabetes, infection, and inflammation) and to the requested treatment, that is oxygen supplementation, mechanical ventilation, surfactant therapy (29). Lung injury in RDS may start as acute inflammatory changes due to FRs, such as focal hypertrophy and altered metabolic activity of pulmonary endothelial cells as well as inactivation of the small amount of surfactant produced, which may then evolve into chronic lung disease. The risk of chronic lung injury is high especially in very preterm newborns, which frequently require respiratory support through oxygen administration and/or mechanical ventilation to be successfully stabilized. The postnatal increase in oxygen availability contributes to the rapid formation of FRs which exceed the detoxification capacity of the newborn's anti-oxidative defense systems (30), leading to the damage of a variety of cellular components including proteins, lipids and nucleic acid that may result in cell death.

### BPD

BPD is the most frequent cause of chronic lung disease in infancy (31) and should be considered in any newborn requiring oxygen treatment for at least 28 days (32). Lung damage in infants with BPD consists of an arrested acinar development resulting in decreased alveolar number and arterial counts associated with a vascular dysfunction characterized by marked angiogenesis and abnormal distribution of alveolar capillaries with variable vessel density in adjacent alveoli or vessels that are more distant from the air surface. Multiple etiologic factors contribute to permanent lung damage in BPD, such as genetic predisposition, baro- and volutrauma from mechanical ventilation in surfactant-deficient lungs, pulmonary edema, pre- and postnatal infections, as well as generation of FRs from prolonged oxygen use at high concentrations. Damage to respiratory epithelium and endothelium occurs by necrotic/apoptotic cell death followed by resolution of injury, with impaired alveolarization and angiogenesis resulting in fewer, larger alveoli and dysmorphic pulmonary vasculature. In particular, inadequate angiogenesis is an important component of the mechanism of BPD, leading to the dysplasia of blood vessels and alveoli. Infants with BPD show a decreasing tendency for VEGF production, which plays a crucial role in the remodeling and repair of lung injury during hyperoxia- induced injury of the microvasculature. The decreased expression of VEGF results in the cessation of sprouting during lung angiogenesis, leading to a characteristic block of blood vessels and a stalled alveolus in BPD. Intrinsic to the physiopathology of BPD is prematurity through altered lung development, immature antioxidant systems, inadequate nutrition and need for oxygen supplementation and/or mechanical ventilation. In addition, it has been demonstrated that OS inhibits pulmonary surfactant function by lipid peroxidation and damage of surfactant proteins. The enzyme glycerol-3-phosphate acyl-transferase, which catalyzes the first reaction in phosphoglyceride synthesis, is very sensitive to oxidative damage and type-II cells exposed to FRs show a decreased phosphatidylcholine synthesis. Surfactant proteins are a target for FRs too. In particular, oxidized SP-A loses its surfactant and immune defense functions. Inactivation or disturbed function of surfactant may therefore play a significant role in the pathogenesis of BPD (33).

The role of OS has been proved in RDS, the most frequent prematurity-related disease, as well as in the development of BPD (29). In this context, the oxidative damage occurring in lipids, proteins, and DNA has been investigated in many clinical studies over the last decade, mainly involving preterm newborns.

### Biomarkers of OS in RDS

In cord blood of preterms who develop RDS (mean gestational age, GA, 29.6 ± 1.5 weeks) it has been shown a significant increase of MDA (*p* = 0.004), protein carbonyl (*p* = 0.004) and 8-hydroxy-2-deoxy guanosine (8-OHdG, *p* = 0.021) with respect to healthy controls, suggesting that neonatal RDS is characterized by damage of lipids, proteins, and DNA, as a consequence of OS (34). In a later study, significant higher plasma levels of protein carbonyls and oxidant/antioxidants ratio (protein carbonyls/{Superoxide Dismutase + Glutathione Peroxidase}) were found in preterms with RDS (mean GA 31.41 ± 2.31 weeks) compared with healthy controls (*p* < 0.001). Interestingly, birth weight and gestational age were negatively correlated with both plasma protein carbonyls and oxidant/antioxidants ratio, whereas non-significant correlations were found between the levels of OS markers and the severity of RDS (35). The role of OS in neonatal lung injury has been confirmed in a study showing a significant increase of MDA and H_2_O_2_ plasma levels (*p* < 0.005) in preterms with RDS (mean GA 31.2 ± 3.2 weeks) in comparison with healthy controls (36). Recently, Elkabany et al. (37) demonstrated that serum birth levels of MDA, AOPPs, and 8-OHdG were significantly higher (*p* = 0.031, *p* < 0.001, *p* < 0.001, respectively) in newborns with RDS than controls, with a further increase after 3 days (*p* < 0.001). Of note, day 0 levels of 8-OHdG and AOPPs were significant independent variables related to RDS severity (*p* = 0.008 and *p* = 0.006, respectively) (37).

### Biomarkers of OS in BPD

A prospective study by Fabiano et al. (38) investigated bronchoalveolar lavage samples collected from preterm infants with RDS, measuring LOOH concentrations in those who developed BPD (mean GA 26.9 ± 1.9 weeks) and in controls without BPD. LOOH levels were significantly higher (*p* < 0.01) in the BPD group (median 16.35 nmol/mL) than in controls (median 13.18 nmol/mL). A significant correlation between BPD occurrence and LOOH concentrations (*p* < 0.05) was observed, suggesting that early biochemical monitoring of preterms with RDS might predict the development of BPD (38). In a recent cohort study on very low birth weight preterm infants, 8-OHdG concentrations in serum and tracheal aspiration (TA) samples on the 1st day after birth resulted significantly higher in those who subsequently developed BPD than in the non-BPD group (*p* < 0.05). The 8-OHdG levels in TA fluid were still found significantly higher in the BPD group than in controls at day 28 (*p* < 0.05). Furthermore, TA 8-OHdG levels (cutoff, 4.4 ng/mg) showed a sensitivity of 81.5% and a specificity of 64.4% in predicting BPD occurrence. These findings provide evidence that oxidative DNA damage in the respiratory tract may be involved in the development of BPD (39). Biomarkers of OS-related damage could be useful in patients with BPD in order to early detect lung infectious diseases that may worsen their clinical course. In mechanically ventilated newborns with BPD, surveillance of nosocomial lung infection is crucial, given the associated high morbidity and mortality (40). Interestingly, 3-chlorotyrosine (Cl-Tyr), an AOPP formed by the reaction between hypochlorous acid (HOCl) produced by the neutrophil enzyme myeloperoxidase and tyrosine residues in proteins, showed a fair ability in detecting bacterial growth in endotracheal aspirates from ventilated preterm newborns <32 weeks GA. Therefore, this marker of OS-related protein damage could be promising for monitoring OS and lung infection status in these patients (41).

According to the aforementioned studies, prematures with RDS, as well as those who will develop BPD, show significant differences in the oxidation of lipids, proteins, and DNA when compared to healthy newborns. These findings suggest that OS contributes to cellular and molecular changes that may impair lung growth and development. There is also evidence that the observed differences in oxidation patterns are ascertainable in the first few days of life, indicating that the pathological process of OS-related lung injury occurs early after birth. In spite of the advances achieved in the perinatal and postnatal management of preterm newborns, the incidence of both RDS and BPD still shows a flat trend (42). Therefore, the availability of quantitative parameters of OS-related lung damage at the subclinical stage could be particularly valuable in the clinical management of these patients for targeting early interventions that might contribute to improve short and long-term health outcomes.

## Biomarkers of Oxidative Damage in the Newborn Lung: Evidence Gaps and Limitations

The development of OS biomarkers has gained increasing research interest since they may provide useful insights about physiopathological pathways underlying OS-mediated pulmonary damage in newborns. In this context, biomarkers obtained from biomolecule oxidation could be defined as metabolites detected in biosamples able to evaluate host susceptibility to OS by measuring lipid, protein, and DNA damage. Their implementation in clinical settings may help to early identify high risk-newborns, diagnose OS-associated lung disease and provide targeted treatment. Biomarkers could be also useful for monitoring the pharmacologic response to antioxidant interventions.

Among biomarkers derived from lipid peroxidation, MDA is one of the most used as a marker of oxidative damage in the newborn lung through the thiobarbituric acid reactive substances (TBARS) assay. However, MDA is not exclusively derived from polyunsaturated fatty acids and most of the TBARS are not related to lipid oxidation, so the measurement of this biomarker should be interpreted with caution (43). LOOH are specific products of lipid peroxidation which may accumulate in several pathological conditions, including chronic pulmonary diseases such as cystic fibrosis (44) and asthma (45). They can be measured by spectrophotometric assay and their use would be of interest to investigate OS-related lung injury in newborns at risk for chronic lung disease. Promising biomarkers of lipid peroxidation are prostaglandin-like compounds such as IsoPs and IsoFs, which can be easily measured by means of gas chromatography coupled to mass spectrometry (GC-MS) technique, liquid chromatography coupled to MS (LC-MS) and immunological assays (1). With regard to protein oxidation biomarkers, carbonyl proteins have been frequently used to investigate OS-related lung injury in newborns, thanks to their relative stability and the availability of various detection methods such as spectrophotometric assay, enzyme-linked immunosorbent assay (ELISA), and Western blot immunoassay (46). A further marker of OS-related protein damage is represented by AOPPs, which can be measured using a spectrophotometric assay. However, this test showed poor reproducibility and advanced methods are required in order to obtain more reliable results (47). 8-OHdG has been the most commonly used biomarker of DNA oxidative damage in the newborn lung due to its reliability and relative stability; it can be easily detected in different biosamples through available methods such as ELISA and LC-MS (5). A summary of the most frequently used biomarkers of OS for neonatal lung diseases obtained from biomolecule oxidation is shown in Table 1.

**Table 1:** Summary of the most frequently used biomarkers of OS-related lung damage in the newborn.

**Biomarker**	**Biosamples**	**Analytical methods**	**Neonatal disease**	**References**
**Lipid peroxidation**				
MDA	Cord blood; newborn blood	TBARS assay	RDS	(34, 36, 37)
LOOH	BAL	Spectrophotometric assay	BPD	(38)
IsoFs	Urine	GC-MS/MS; UPLCMS/MS	BPD	(49, 50)
**Protein oxidation**				
Carbonyl proteins	Cord blood; newborn blood; TA	Spectrophotometric assay; immunological assays	RDS	(34, 35)
AOPPs	Newborn blood	Immunological assays	RDS	(37)
**DNA oxidation**				
8-OHdG	Cord blood; newborn blood; TA, urine	Immunological assays	RDS, BPD	(34, 37, 39, 51, 52)

*8-OHdG, 8-hydroxi-2′-deoxiguanosine; AOPPs, advanced oxidation protein products; BAL, bronchoalveolar lavage; BPD, bronchopulmonary dysplasia; IsoFs, isofurans; GC, gas chromatography; LOOH, lipid hydro-peroxide; MDA, malondialdehyde; MS/MS, tandem mass spectrometry; RDS, respiratory distress syndrome; TA, tracheal aspirate; TBARS, thiobarbituric acid reactive substances; UPLC, ultra-performance liquid chromatography*.

Ideally, a biomarker should demonstrate ease of use, biological validity and reproducibility, high sensitivity and specificity. Furthermore, the ideal biomarker should require a small sample volume and should be preferably obtained by non-invasive automatized procedure with immediate results (48). Most of the studies published so far evaluated OS-biomarkers in blood samples, whereas a few used TA and bronchoalveolar lavage samples. However, these invasive procedures of sampling are not suitable for monitoring evolving conditions over time. Therefore, there is an increasing need for non-invasive procedures for monitoring biomarkers with desired frequency and without causing pain or distress. Urine is a non-invasively obtained biosample that can be easily used for measuring biomarkers of OS-related lung damage in newborns (4). Urinary isofuran levels have been significantly correlated (*p* < 0.01) with the development of BPD in newborns of 24–28 weeks of GA (49). More recently, Kuligowski et al. (50) demonstrated that the urinary concentration of IsoFs in the first 4 days after birth correlated with later development of BPD in preterm infants <32 weeks of GA. Joung et al. (51) demonstrated that the 8-OHdG urinary levels on the 7th day of life were an independent risk factor for developing moderate/severe BPD in preterms with GA <30 weeks (51). Later, significant differences in urinary levels of 8-OHdG during the early postnatal period (*p* < 0.05) were observed when comparing preterms with no-or-mild BPD and those with moderate-to-severe BPD (52). Taken together, these results provide evidence that early urinary determination of lipid and DNA peroxidation byproducts may be useful for the evaluation of OS-derived lung damage in newborns at risk for developing chronic lung disease. Thus, urine could be considered a useful matrix for reliably detecting different oxidized products. For this purpose, also saliva samples seem promising although their use still requires validation before application in clinical practice (4).

With regard to the analytical methods employed so far, most are based on immunoassays and/or spectrophotometry. These methods are easily applicable but their implementation is limited by poor sensitivity and specificity and still needs validation in clinical practice (10). Advanced methods based on mass spectrometry determination show the advantage of high selectivity and sensitivity. Novel options for non-invasively detecting the pulmonary OS-related damage are reproducible and validated techniques such as the electronic-nose and Nuclear Magnetic Resonance spectroscopy of the exhaled breath condensate (9). To help confirm lung damage, Sepehr et al. (53) previously demonstrated the utility of optical cryoimaging for measuring mitochondrial redox state by an automated image acquisition and analysis system able to acquire fluorescence images of lung tissue sections in neonatal rats. Moreover, X-ray tomography and imaging of lung sections combined with genetic labeling were used to develop static, 3-dimensional pictures of alveologenesis for a full representation of the cellular architecture in murine lungs (54, 55). Nonetheless, the use of such techniques has not been validated *in vivo* studies and is still not suitable in the clinical practice, also due to the complex technical procedures as well as the high costs (4). The development of innovative analytical methods with greater clinical applicability may hopefully contribute in the upcoming future to early identify newborns at higher risk of lung injury and to shape targeted approaches for prevention and treatment of the disease. Indeed, only few of the biomarkers developed so far have been qualified for neonatal lung disease and their analysis has been restricted to research settings.

To this regard, a previous study in a murine model of BPD suggested a possible set of differentially expressed proteins in BPD (i.e., p-AMPKb1[S108], platelet-derived growth factor receptor PDGFRb, and SLUG) based on their relevance in developmental lung injury and such set was further validated on a cross-platform transcorrelation analysis (protein/transcript) (56). However, this kind of studies are still preliminary and present several limitations. First, the mouse lungs at birth are functionally different than those of a preterm infant and a murine model of hyperoxic lung injury may not accurately model human BPD. Second, omics analysis at single time-points may not identify all dysregulated genes, proteins, and pathways.

Last but not least, the translation into clinical practice of the findings obtained in the small clinical studies published so far is limited by the lack of reliability and consistency of biomarkers and the lack of statistical power. Overall, the available pieces of evidence underline the need for further research on a large scale and over longer monitoring periods to obtain more robust results and to allow serial determinations of OS biomarkers.

## Conclusions

The role of OS in neonatal lung injury is complex and likely not thoroughly understood. Despite emerging evidence in research settings, currently no one biomarker is able to definitely and accurately predict lung damage in newborns. Therefore, the current clinical practice is limited by a lack of ability to distinguish very early those newborns who will likely develop chronic lung disease. Detection and monitoring of OS-related lung damage through non-invasive determinations of different oxidized products would be highly advantageous in clinical settings. With regard to BPD, the determination of 8-OHdG in urine proved a fair ability in discriminating healthy preterm from those with BPD (51), as well as in distinguishing preterms with no-or-mild BPD from those with moderate-to-severe BPD (52). Moreover, urinary isofurans are promising non-invasive biomarkers for BPD prediction (49, 50). Nonetheless, even though an increasing amount of data supports the role of OS, it seems that the complexity of this multi-factorial condition might not be captured by a single biomarker. Instead, researchers should move toward the development and validation of suitable panels of biomarkers that more reliably may predict such health outcome. Early diagnosis and treatment of OS-associated lung diseases may be crucial to prevent adverse outcomes that can extend beyond the neonatal period, through impaired lung growth and function that may increase the risk of respiratory diseases later in life.

## Author Contributions

GF and MG: conceptualization. GF, GC, and GL: writing original draft. MG: review and editing. All the authors read and approved the final version of the manuscript.

## Conflict of Interest

The authors declare that the research was conducted in the absence of any commercial or financial relationships that could be construed as a potential conflict of interest.
